# Poster Session I - A41 TESTING THE EFFECTS OF TIME RESTRICTED FEEDING IN COLORECTAL TUMORIGENESIS

**DOI:** 10.1093/jcag/gwaf042.041

**Published:** 2026-02-13

**Authors:** R Patel, V Carmona-Alcocer, A Mcbride, P Karpowicz

**Affiliations:** Biomedical Sciences, University of Windsor, Windsor, ON, Canada; Biomedical Sciences, University of Windsor, Windsor, ON, Canada; Biomedical Sciences, University of Windsor, Windsor, ON, Canada; Biomedical Sciences, University of Windsor, Windsor, ON, Canada

## Abstract

**Background:**

Circadian rhythms, governed by the circadian clock, regulate many daily physiological processes such as stress responses, inflammation, metabolism, cell differentiation, and stem-cell maintenance and regeneration. The loss or disruption of circadian rhythms increases intestinal tumorigenesis in mouse models, while intestinal tumours are thought to have a dysfunctional molecular clock in both human and mouse-based studies.

Time Restricted Feeding (TRF), a dietary regimen where feeding is restricted to active periods, can strengthen and synchronize circadian rhythms; pilot experiments in our lab revealed that TRF reduces intestinal tumorigenesis, but the mechanism behind this effect is unknown.

**Aims:**

Using the Apc^min^ mouse model of colorectal cancer, that recapitulates the loss of the tumour suppressor gene *Apc* that occurs during the incipient stages of colorectal tumorigenesis, we explored whether TRF functions by reducing tumour growth or initiation.

**Methods:**

A multi-week TRF regimen was performed on sex and aged matched Apc^min^ mice, restricting feeding to the night when activity is at its peak. Small intestinal tumour number quantification was performed to test tumour number differences between TRF and ad libitum fed control groups (food access = 24h per day). Histopathology was used to identify microscopic tumours, and to measure tumour area to test tumour initiation and growth.

**Results:**

Large tumour number is reduced by TRF, but histology revealed that relative area and number of microscopic tumours was the same between TRF and control groups.

**Conclusions:**

TRF reduces large tumour number relative to controls, but histological analysis revealed that tumour initiation and growth is the same between TRF and controls. Further histopathological analysis and single-cell RNA sequencing will test whether TRF changes cell survival, protein translation, and circadian clock activity.

**Funding Agencies:**

CIHRWorldwide Cancer Research, and the Cancer Research Society.

